# Artificial intelligence, complexity, and systemic resilience in global governance

**DOI:** 10.3389/frai.2025.1562095

**Published:** 2025-06-02

**Authors:** Andrés Ilcic, Miguel Fuentes, Diego Lawler

**Affiliations:** ^1^Facultad de Filosofía y Humanidades, Universidad Nacional de Córdoba, Córdoba, Argentina; ^2^Instituto de Investigaciones Filosóficas, SADAF–CONICET, Buenos Aires, Argentina; ^3^Santa Fe Institute, Santa Fe, NM, United States; ^4^Instituto de Sistemas Complejos de Valparaíso, Valparaíso, Chile

**Keywords:** artificial intelligence (AI), systemic resilience, complexity science, international governance, ethical AI, socio-technical systems, adaptive governance, science diplomacy

## Abstract

Artificial Intelligence (AI) is reshaping international governance, presenting opportunities to enhance systemic resilience while posing significant ethical, social, and geopolitical challenges. This paper argues that complexity science offers a valuable framework for navigating AI's integration into global governance systems. We analyze AI's dual capacity as both a transformative tool for improving decision-making, resource allocation, and crisis management, and as a disruptive force introducing risks like data bias, exacerbated inequalities, and governance gaps. By framing resilience as a crucial, boundary concept bridging disciplines and practice, we advocate for adaptive, inclusive governance models capable of managing the inherent uncertainties of AI-driven complex socio-technical systems. Integrating complexity insights with principles like institutional modularity and robust stakeholder collaboration is vital for fostering equity, accountability, and sustainability. This study proposes a conceptual approach aiming to align technological innovation with societal values, ensuring AI deployment contributes to a more resilient and equitable global future, while at the same time it proposes complexity as a boundary concept to bridge the gap between governance literature and philosophy of science and technology.

## 1 Introduction

Artificial intelligence (AI) is profoundly transforming the technological landscape, creating unprecedented opportunities to enhance operational efficiencies while reshaping social and political frameworks (Binns, 2018; Brynjolfsson and McAfee, 2017). This paper conceptually explores how principles from complexity science can inform the development of resilient governance structures for AI, assessing both its potential benefits and challenges within international systems. We focus on the interplay between governance needs, regulatory frameworks, and complexity science to understand how AI can bolster systemic resilience while addressing critical ethical, social, and geopolitical concerns, bringing analytical categories and perspectives that have been developed within the realm of philosophy of science and technology. As AI technologies become increasingly accessible across universities, computational labs, and consumer markets, they facilitate the creation of digital tools capable of extensive data capture and decision-making (Floridi, 2019; Pasquinelli and Joler, 2021). These advancements, often built on open-source software and delivered via accessible platforms (SaaS), enhance the adaptability of information systems (Stahl and Wright, 2018). This integration promises streamlined processes and improved cross-border collaboration, potentially enabling nations and international agencies to respond more effectively to global challenges and crises (Mayer-Schönberger and Cukier, 2013). This, in turn, may strengthen the resilience capacities of states. However, realizing these benefits necessitates a critical understanding of both AI's technical capabilities and the socio-political complexities it entails when considered as the radical form of technology it is (Harari, 2024).

Systemic resilience, viewed through the lens of complexity theory, provides a foundation for integrating advanced technologies into already very complex systems, encouraging a holistic comprehension of system interactions that support—and require—adaptive and responsive strategies amid emerging challenges. Integrating AI for systemic resilience requires addressing not only technical obstacles but also ethical considerations, governance structures, and societal values. Among others, Coeckelbergh (2025) highlights that while AI can enhance global dynamics, it is imperative to critically assess the implications and limitations of such technologies as they evolve.

The narrative positing that AI inherently enhances systemic resilience often neglects substantial concerns, including data privacy issues, ethical dilemmas, power redistribution, and exacerbated inequalities (Eubanks, 2018), a narrative that has been to a considerable extent put forward by the private companies reshaping the digital infosphere. The reliance on AI-driven digital tools may disproportionately empower those who control these technologies, marginalizing diverse voices in policy-making processes (Noble, 2018). Furthermore, assumptions about seamless cross-border collaboration can be overly optimistic. Geopolitical tensions and divergent national interests frequently impede cooperative efforts, resulting in fragmented rather than unified responses to global challenges (Wong, 2021; Zuboff, 2023). While AI can augment decision-making, the complexities of international relations cannot be effectively managed by algorithms alone. Over-reliance on technological solutions risks diverting attention from root causes of pressing global issues, such as security and economic disparities, potentially exacerbating these challenges (Hulme, 2009).

Recognizing these dynamics, this paper emphasizes that systemic resilience in the global order is not solely a matter of technological advancement or technical fixes. It also requires the development of inclusive governance structures that prioritize equity, justice, and accountability, while integrating technology in ways that meaningfully advance these objectives (Jasanoff, 2004; Mulgan, 2023). Although AI can offer powerful tools for analysis and efficiency, it should not be misconstrued as a universal panacea for the multifaceted challenges of international governance—nor, for that matter, for any other domain of human endeavor. Ethical considerations and epistemic judgments—embedded within a complex tapestry of institutions and political perspectives—remain central to crafting policies that are both effective and equitable, ensuring that diverse voices from across global communities are heard and represented. These are the kinds of policies we consider resilient: those that enhance the systemic functional capacity of the structures they are designed to influence.

The primary aim of this paper is therefore to propose the scaffoldings for a conceptual framework that elucidates how technological advancements and human values can be synergistically integrated to build a more resilient global system. This framework emphasizes collaborative efforts between technological innovation and societal values in addressing complex international challenges. It examines the obstacles and opportunities presented by recent developments in general-purpose technologies, such as AI, drawing insights from diverse disciplines that employ complexity theory and systems thinking (Page, 2010, 2018). By promoting a nuanced understanding of socio-economic ramifications, this multidisciplinary approach seeks to advance the philosophical, conceptual values that guide decisions within both the realm of public policy and corporate management, as a seed to work toward equitable solutions that benefit all sectors of society (Cairney et al., 2019).

The first section explores how AI holds significant operational potential for enhancing global resilience, while acknowledging that its direct integration into societal structures requires careful consideration of ethical, social, and geopolitical factors. A second section explores how the core factors of AI as an innovation driver can better be delineated when considering AI as a series of enmeshed complex systems that require a better model of what the system is and how to govern it by acknowledging the uncertainties involved. A brief third section characterizes current global trends in AI governance in terms of their resiliency impact for the rest of society. Then in the fourth section, we delineate how better governance dynamics can make use of such a conceptual approach for better societal outcomes. By adopting a complexity science-informed approach to governance, this paper aims thus to contribute to the development of strategies that not only leverage technological innovations but also uphold fundamental human values in the face of evolving international challenges, seeing powerful technologies as core tools by means of which a more resilient world-system can be developed for the benefit of all if their design and deployment are guided by the right understanding of the kind of system they are and the ones they modify.

## 2 Systemic resilience in an AI-driven world

Current AI trends are fundamentally reshaping informational dynamics on a global scale, transforming the landscape of international governance both directly and indirectly. Directly, AI enhances what governance structures can achieve; indirectly, it influences geopolitics and fosters the emergence of new global actors. This transformation offers opportunities to bolster systemic resilience while also posing significant challenges that demand attention. While the full articulation will be developed in the final section of the paper, it is important to clarify from the outset that we adopt a broad conception of “governance,” aligned with the framework proposed by McGinnis (2011): “a process by which the repertoire of rules, norms, and strategies that guide behavior within a given realm of policy interactions are formed, applied, interpreted, and reformed” (McGinnis, 2011, p. 171).

The concept of resilience, initially rooted in ecological studies (Holling, 1973) and later expanded into socio-ecological frameworks, has become crucial for addressing interconnected and dynamic global issues—particularly as AI deployment accelerates across societal levels. In the context of global systems and international relations, achieving resilience necessitates a comprehensive understanding of the world system that extends beyond traditional academic disciplines to include principles of complexity, interconnectivity, and adaptability (Chandler, 2014).

Within international relations, systemic resilience describes how effectively the global governance framework—comprising states, institutions, and both formal and informal networks—can withstand disruptions, manage crises, and adapt to emerging challenges while maintaining its core functions and cohesion. A critical component of this framework is its capacity for adaptation and transformation in response to both predicted and unexpected challenges. For resilience to be truly effective, the governance structure itself must be capable of modifying its norms, institutions, and policies as new information and conditions arise. This adaptability aims not only at survival but also at sustainable transformation aligned with long-term objectives.

Assessing contemporary advancements in artificial intelligence through the lens of systemic transformation reveals its unprecedented capacity to permeate all strata of society, presenting both considerable challenges and unique prospects for reshaping global dynamics toward collective wellbeing and addressing extant systemic deficiencies. Abstractly framed, the central issue lies in the imperative of AI governance at the global level (Bullock, 2024; Chinen, 2023). As both scholarly discourse and ongoing public debates underscore, virtually no domain currently leveraging digital technologies remains immune to AI's transformative potential. This observation lends a dual significance to the notion of “global”: AI's impact is not merely geographically extensive but also fundamentally cross-sectoral in scope. Given the breadth and inherent complexity of this issue, a multidisciplinary approach becomes essential to comprehensively grasp the full spectrum of stakes involved.

The perspective we seek to contribute to this debate stems primarily from epistemological reflections on science and technology, with particular emphasis on their entanglement with political economy—conceived here as a complex system (Mulgan, 2023). Emphasizing the epistemological implications of complexity theory is particularly fruitful, as it illuminates the tension between our condition as finite epistemic agents and the ever-evolving, contingent world we continuously reshape through our cognitive and technological interventions.

In this context, the notion of adaptive governance—initially developed to inform policy-making in natural and ecological systems—emerges as a particularly pertinent conceptual tool. At its core, adaptive governance emphasizes the dynamic interplay between formal and informal institutional networks, processes of social learning, and sustained community engagement (Akther and Evans, 2024; Folke et al., 2005). These elements work synergistically to strengthen governance architectures, enabling more responsive and context-sensitive forms of environmental management. Moreover, this framework proves especially valuable in socio-ecological systems across the Global South, where structural vulnerabilities and historical asymmetries render the implementation of resilient and inclusive governance models both more urgent and more complex.

From our perspective, systemic resilience stands as a foundational attribute of both natural and artificial ecosystems. It ought to be envisioned not merely as a desirable outcome but as a core design principle underpinning adaptive governance structures—particularly in contexts marked by high complexity and persistent uncertainty. Resilience, understood as the capacity of a system to absorb shocks, adapt to perturbations, and reorganize without forfeiting its essential functions, proves indispensable not only in ecological settings but also in increasingly interdependent socio-technical systems. In such environments, decision-making must remain agile, reflexive, and robust, capable of withstanding volatility while maintaining coherence and responsiveness.

Resilience thinking, therefore, offers a conceptual lens through which to understand the intricate interdependencies that characterize social, economic, and environmental systems. In the context of our inquiry, it proves particularly instructive for examining how AI-driven innovations reshape governance architectures and influence global systemic dynamics. Crucially, it also helps identify potential leverage points—or control surfaces—for enhancing institutional adaptability and performance. Davidson et al. (2016) offer a thorough review of resilience across disciplinary boundaries, developing a taxonomy that clarifies how systems operating at different scales respond to stress through adaptation and transformation. Their analysis underscores the utility of resilience as a heuristic for policy design by promoting interdisciplinary approaches to sustainable solutions, provided sufficient conceptual clarity is achieved and maintained.

Nisioti et al. (2023) advance an information-theoretic framework that significantly sharpens the conceptual contours of resilience. Their contribution lies in articulating a typology that distinguishes four forms of resilience, predicated on whether systems preserve or transform their structural and functional properties in response to perturbations. This classification yields an operational lens through which resilience can be applied across heterogeneous domains, conceptualizing it as the capacity of systems to sustain functional adequacy—i.e., fitness—through diverse adaptive strategies. Crucially, they position resilience as a “boundary concept,” one that facilitates epistemic translation and interdisciplinary exchange, thereby equipping both researchers and practitioners with a shared conceptual tool for navigating complexity within their respective contexts.

Conceiving resilience as a boundary concept enables stakeholders to navigate across disciplinary and institutional divides, fostering integrative dialogues between diverse epistemic communities and practical domains. This interpretive flexibility allows resilience to function as a common reference point, facilitating the co-production of knowledge and the design of governance mechanisms attuned to complex, dynamic environments. Such an approach supports the development of adaptive institutional capacities capable of responding to global challenges—climate change, economic volatility, and geopolitical tensions among them—that both shape and are shaped by the deployment of artificial intelligence.

In the realm of international relations, artificial intelligence exhibits considerable potential for reinforcing systemic resilience, particularly through its deployment in disaster response, conflict forecasting, and global health surveillance. These applications enable more anticipatory and adaptive approaches to crisis management, thereby strengthening the responsiveness of global governance systems (Essien and Petrounias, 2022; Cao, 2023). Yet, the integration of AI into these domains also surfaces profound ethical dilemmas and governance complexities—especially when situated within the geopolitical dynamics of the ongoing “AI race” (Kissinger et al., 2024; Naudé and Dimitri, 2020).

This global competition among states, corporations, and research institutions for technological supremacy underscores the strategic significance of AI, while simultaneously raising urgent concerns regarding algorithmic bias, privacy infringements, and the unequal distribution of technological benefits. Prominent policy frameworks—such as China's “AI Superpower” initiative, the European Union's coordinated AI strategy, and recent executive orders in the United States—attest to the scale and intensity of state-level investments in this domain. In parallel, technology companies like Nvidia, Google, and Microsoft, alongside a growing constellation of startups, continue to drive innovation at a breakneck pace—often privileging speed and market dominance over safety, transparency, and regulatory alignment (Hartmann and Henkel, 2020; Bessen et al., 2018).

This competitive development environment generates a fundamental paradox: while it accelerates innovation, it simultaneously undermines systemic resilience by exacerbating global inequalities and bypassing crucial regulatory safeguards. Nations with advanced technological infrastructures are increasingly shaping the landscape of global governance, often marginalizing less developed countries and reinforcing asymmetrical dependencies (Rogers et al., 2023; Maas, 2021). Addressing these challenges requires governance frameworks that not only integrate resilience as a central design principle but also prioritize inclusivity, equity, and ethical accountability.

Recent scholarship has put forward a variety of responses to the governance challenges posed by systemic risks. Bouckaert and Galego (2024) highlight the necessity for institutional reform, advocating for models such as the New Weberian State and Whole-of-Government approaches to enhance coordination and responsiveness during crises. Renn et al. (2022) stress the importance of balancing efficiency with resilience, particularly in the context of climate change and pandemic management. Their work underscores the need for risk-informed governance frameworks that extend beyond technocratic efficiency to include participatory mechanisms and deliberative input. In a similar vein, Schweizer and Juhola (2024) advocate for inclusive governance models that acknowledge the plural nature of risk perception and prioritize stakeholder engagement in decision-making processes.

AI's dual role—as both a tool to address disruption and a potential source of it—places it at the heart of resilience governance. Its ability to enable real-time data analysis, optimize resource allocation, and inform adaptive policy design holds transformative potential for reshaping global governance frameworks.

Machine learning algorithms have the capacity to identify conflict signals and predict the impacts of disasters, thereby supporting preemptive decision-making (Arias-Vargas et al., 2024; Harriott, 2024). However, these advancements also carry the risk of exacerbating inequalities if not accompanied by robust oversight mechanisms and collaborative initiatives aimed at democratizing AI resources. Resilience frameworks informed by complexity science, such as those proposed by Schweizer and Juhola (2024), stress the importance of adaptive and inclusive governance in addressing systemic risks.

A governance paradigm rooted in resilience must engage with both the ethical and epistemological dimensions of AI deployment within complex systems. Chandler's (2014) critique of neoliberal resilience frameworks highlights the necessity of embracing emergent complexity in policymaking, advocating for governance models that align technological design with societal needs—something that cannot be achieved through market forces alone. This perspective ensures that AI-driven solutions tackle root causes rather than merely offering superficial technical fixes, as cautioned by Huesemann and Huesemann (2011) and Klein (2014) in the context of climate change policy.

If this analysis holds, enhancing systemic resilience through AI requires robust global cooperation and the establishment of ethical standards that prioritize fairness, transparency, and accountability. This imperative calls for the active involvement of international organizations in aligning national regulatory frameworks and in fostering transnational knowledge-sharing mechanisms. Such initiatives are crucial not only to ensure equitable access to advanced AI technologies but also to safeguard their responsible use—especially in contexts where existing disparities in capacity and influence threaten to exacerbate structural inequalities. Building truly resilient governance systems demands inclusive frameworks that empower all stakeholders, particularly marginalized communities, while addressing the challenges posed by accelerating technological change and emerging epistemic challenges. As nations and global actors navigate the transformative landscapes shaped by AI, the way in which the balance between innovation, equity, responsibility, and sustainability is negotiated will determine the future trajectory of global governance. To ensure that this balance is resilient to unforeseen challenges, the conceptual-theoretical foundation must begin by recognizing AI as a complex system that permeates existing socio-technical structures; this will be explored in the next section.

## 3 Conceptualizing artificial intelligence as a complex system

The integration of AI into already complex systems introduces a degree of unpredictability and complexity that traditional models of society and technological development are ill-equipped to address. As AI systems grow in capability, their interconnections with other technologies can generate effects that are opaque and challenging to disentangle in real-world contexts. This complexity calls for a revaluation of our problem-solving and decision-making frameworks, necessitating the adoption of new methodologies that can accommodate the dynamic, often non-linear nature of AI-driven systems.

This suggests that AI should be conceptualized and treated as a complex system. The pervasive role of artificial intelligence within modern society calls for a reassessment of its nature and consequences through the lens of complexity science. Traditional reductionist scientific approaches, which isolate discrete components for analysis in idealized conditions, fail to capture the intricate interdependencies and emergent behaviors that characterize AI systems. Treating AI as a complex system offers a more productive perspective, one that not only clarifies its multifaceted dynamics but also facilitates the alignment of its development with societal resilience. When guided by the appropriate values and supported by robust institutions, AI can become a transformative force contributing to societal resilience. This section delves into AI as a complex system, highlighting the crucial role of boundaries, the interdependence of socio-technical networks, and the inherent uncertainties that shape its design, deployment, and governance—factors that collectively necessitate its treatment as a complex system.

Complexity science offers a multidisciplinary framework for understanding systems that are characterized by non-linearity, feedback loops, and emergent properties—traits that defy the traditional linear and hierarchical models commonly used in technological and policy analyses. A central concept in this approach is that of boundaries, which delineate the scope and influence of interactions within a system (Holland, 2012). In socio-technical systems, these boundaries are not fixed; they are fluid constructs, shaped by historical contexts and the perspectives of various stakeholders. As these systems evolve, so too do their goals, creating dynamic shifts that reflect the changing nature of the system itself. The permeability of these boundaries is a critical factor in shaping the interactions between system components, as well as the overall resilience of the system. This makes boundaries a key focal point for the study and design of complex systems, particularly when considering how they adapt and respond to new agents and decision-dependent structures (Simon, 1996).

A central reason for highlighting Nisioti et al.'s (2023) characterization of resilience as a “boundary concept” in the previous section lies in its significant methodological value for policy design. Boundary concepts prove especially beneficial in contexts that require interdisciplinary collaboration, as they offer operationalizable frameworks that facilitate communication across specialized domains. This communicative function is essential when addressing the continuously shifting interfaces between technological and social systems. The dynamic nature of these boundaries mirrors the ongoing reciprocal relationship between emerging technologies and the societal structures they both influence and are influenced by, as exemplified by AI. This reciprocity underscores why boundary concepts are instrumental in developing effective policies for AI governance. Moreover, such concepts—and their technological counterparts—can themselves serve as governance tools, particularly because they enhance the modeling of complex systems by fostering collaboration and communication among actors with different forms of expertise. To effectively leverage these concepts, policymakers, stakeholders, and advisory scientists must recognize their epistemic affordances, as well as the inherent limitations of modeling instruments (Bailer-Jones, 2009; Magnani and Bertolotti, 2017).

In the context of AI as a socio-technical system, boundaries delineate the scope and nature of interactions among various entities, such as governments, private enterprises, and civil society. However, these boundaries are increasingly blurred as AI becomes integrated into diverse aspects of life, ranging from healthcare and governance to online shopping recommendation systems and automated software coding. This permeability fosters unprecedented levels of interconnectedness, which, while offering numerous opportunities for innovation and enhancing the potential for systemic resilience, also introduces new vulnerabilities. For example, the real-time data-sharing capabilities of AI systems can significantly improve decision-making processes. Yet, this interconnectedness also exposes societies to systemic risks such as cascading failures or cybersecurity breaches—vulnerabilities that are now easier to identify and exploit (Essien and Petrounias, 2022).

This duality—an inherent feature of all powerful technologies—highlights the need for a nuanced understanding of boundary dynamics through the lens of complexity science. Effective governance, particularly in the context of AI, hinges on the ability to navigate these shifting boundaries and the potential regime changes that can emerge rapidly and unexpectedly. AI systems, along with their societal impacts, exhibit key characteristics of complex adaptive systems, including non-linear dynamics, threshold effects, cascades, and limited predictability. Traditional governance approaches, however, often falter when confronted with these properties, as they tend to assume linear, predictable relationships between policy interventions and outcomes. In response, governance frameworks must evolve to incorporate the inherent unpredictability of AI systems, emphasizing flexibility, adaptability, and the capacity to respond to emerging challenges rather than relying on static models of cause and effect. As Duit and Galaz emphasize in their seminal work on complexity and governance, there is a dual justification for adopting a complexity perspective: “It is not only the policy process that alternates between periods of stability and abrupt change. Many of the systems we try to govern are themselves displaying one or several CAS-like properties” (Duit and Galaz, 2008, p. 317). This crucial insight underscores why conventional governance models—whether state-centric or market-driven—are ill-equipped to address challenges that arise from AI-driven transformations. These models, rooted in assumptions of stability and predictability, fail to account for the dynamic and often unpredictable shifts that characterize complex adaptive systems (CAS). This issue is not isolated to AI alone but extends to broader global dynamics, as highlighted by Chandler (2014), whose critique of neoliberal resilience frameworks we alluded to in the first section draws attention to the limitations of traditional governance structures in dealing with the rapidly changing landscapes of technology, politics, and society; even if said structures took advantage of a notion of resilience. Thus, what seems to be lacking is a complexity-informed governance approach to the very concept of resilience for complex systems, informed by what we know are the intrinsic uncertainties involved in knowing such systems. This is essential to navigate the instability and non-linearity that AI innovation inevitably introduces. Traditional governance models encounter distinct and well-documented limitations when tasked with managing complex adaptive systems (CAS). State-dominated approaches, typically structured as top-down hierarchies, are particularly prone to generating distorted evaluative mechanisms. This is largely due to the systematic degradation of information as it moves upward through bureaucratic layers. Such systems are characterized by decision-blocking junctures and rigid procedural bottlenecks, which restrict both the flow of accurate feedback and the capacity for agile response. These limitations are not merely operational but epistemological: hierarchical governance structures struggle to perceive and process the full scope of systemic complexity, leading to blind spots and delayed adaptation. As a result, their ability to undergo timely and meaningful transformation in response to environmental shifts is significantly impaired. This critique has long served as a cornerstone for advocates of market-led governance, who argue that decentralized systems, by contrast, possess greater inherent adaptability. For societal resilience, a critical limitation of market-led governance lies in the epistemic asymmetries that markets themselves structurally require to function. Markets, by design, reward strategic information retention rather than open disclosure. This incentivizes actors to hoard knowledge, generating persistent asymmetries that systematically disadvantage less informed participants. Such information failures are not incidental—they are constitutive of markets as institutions that valorize competitive advantage over collective transparency. This dynamic leads to the chronic overproduction of socially and environmentally harmful goods—such as polluting technologies or extractive AI systems—and the underproduction of beneficial public goods like accessible healthcare, equitable education, or transparent digital infrastructure. The root of this dysfunction lies in the absence of structural incentives for comprehensive information-sharing among all societal stakeholders. Rather than promoting epistemic inclusivity or deliberative coordination, market mechanisms privilege the narrow valuation metrics of shareholder primacy—typically construed in terms of short-term equity value for owners. Within this framework, transparency becomes a liability rather than a virtue, and the rational utility-maximizing behavior of individual actors—entirely coherent within the institutional logic of markets—produces systemic effects that undermine resilience. In this sense, informational asymmetry is not merely a technical glitch but a deeply entrenched epistemological problem. Addressing it requires rethinking the institutional architectures and value systems that define what counts as “rational” or “efficient” in the first place. Complexity science offers a compelling lens through which to explore middleground solutions—those that avoid the pitfalls of rigid state control and laissez-faire market logic alike. In the context of proliferating AI technologies—which are epistemic technologies par excellence—this approach is especially promising. AI systems, by virtue of their capacity to process, structure, and act upon vast and distributed forms of information, open up unprecedented avenues for reimagining governance architectures. They also necessitate it.

Here, the concept of polycentric governance becomes particularly salient. Rather than relying on a single center of authority, polycentric models distribute decision-making across multiple overlapping institutions, enhancing adaptability, robustness, and responsiveness in the face of uncertainty (see section four below). These features are crucial in an era where societies are increasingly structured as knowledge societies, in which information, innovation, and digital infrastructures become not only central resources but defining characteristics of social life. As socio-technical interdependencies deepen and entrench, the stakes of both positive and negative network effects grow. AI systems can rapidly scale beneficial innovations—like real-time epidemic tracking or environmental monitoring—but can just as easily propagate systemic risks, including algorithmic discrimination or cascading infrastructural failures.

Complexity science provides conceptual tools for understanding and governing these dynamics by foregrounding the role of emergence: the idea that system-level outcomes often cannot be inferred from individual components in isolation. This principle is directly applicable to AI's societal integration, where effects such as information bubbles, automated decision-making regimes, or global labor market reconfigurations emerge not from any one algorithm or actor, but from the interactions among them.

One critical insight from complexity science is the central role of feedback loops in shaping system dynamics. Positive feedback loops can accelerate the adoption of AI technologies by reinforcing adoption incentives, technological capability, and market concentration. Yet these same dynamics can also exacerbate inequalities and reinforce systemic biases if not properly checked (Bessen et al., 2018).^1^

In contrast, negative feedback loops help stabilize systems by dampening or reversing destabilizing trends, thereby maintaining system equilibrium. Recognizing and managing these opposing forces is essential to fostering systemic resilience—a concept that links complexity science and governance theory.

As previously discussed, resilience refers to the capacity of systems to absorb disturbances, adapt to change, and preserve core functions over time. Complexity science offers tools and frameworks to enhance this capacity by acknowledging the interdependent, non-linear, and emergent properties of socio-technical systems. Yet, by enabling these systems to manage complexity, it also exposes them to potential tipping points—situations where minor perturbations can trigger cascading transformations. Such systems are said to operate at the edge of chaos, a critical threshold where adaptive potential is highest, but so is vulnerability.

The relevance of complexity science to AI governance becomes particularly evident in the context of wicked problems, as elaborated by Termeer et al. (2019). These problems are marked by high degrees of complexity, uncertainty, and divergent stakeholder perspectives, making them resistant to linear, technocratic solutions (Ludwig, 2001; Rittel and Webber, 1973). Given AI's capacity to amplify both complexity and ambiguity, a complexity-informed governance approach is not merely advantageous but necessary.^2^

AI governance epitomizes a wicked problem, demanding interdisciplinary collaboration and adaptive strategies responsive to dynamic, uncertain conditions. Methodologies drawn from complexity science—such as agent-based modeling, network science, and data mining—equip policymakers with powerful tools to simulate scenarios, anticipate outcomes, and design interventions calibrated to the non-linear dynamics of socio-technical systems (Johnson, 2015).

Agent-based models, for example, simulate interactions among heterogeneous agents within a given system, allowing researchers to observe emergent patterns that are otherwise obscured in aggregate analyses. In the case of AI governance, these models can illuminate how different policy choices influence technology adoption, social behavior, regulatory compliance, and unintended consequences. Network science, by contrast, focuses on the structure and topology of relationships within and across systems, revealing critical interdependencies, vulnerability points, and diffusion pathways that shape systemic outcomes (Nepelski and De Prato, 2020). These tools do more than deepen our understanding of AI as a complex adaptive system; they help construct governance frameworks that are reflexive, adaptive, and sensitive to real-time change. Notably, AI itself is not merely the object of complexity-informed inquiry—it is also becoming an indispensable instrument for the study of complex systems and wicked problems. As Locklear (2025) argues, the computational power and pattern-recognition capabilities of AI enhance our ability to model, map, and respond to the multifaceted challenges posed by contemporary governance dilemmas.

The interplay between AI and societal resilience underscores the importance of addressing epistemic uncertainties in decision-making processes. Complexity science advocates for a holistic approach that embraces uncertainty rather than seeking to eliminate it. This perspective aligns with Lederach's (1996) notion of reconciliation as a means of fostering dialogue and understanding among stakeholders with divergent interests—originally in extreme civil war scenarios—, a point further scrutinized by Chandler (2014) and that we take to highlight the importance of systemic thinking and soft-system methodologies tailored for human-action-decision loops in different scenarios that are characterized by stakeholders with possibly different values, objectives and risk considerations (Checkland, 1981; Checkland and Poulter, 2006).

Applied to AI governance as a complex issue, this approach underscores the necessity of inclusive and iterative processes that accommodate the plurality of perspectives and values embedded within complex systems—both those that AI is used to model and those within which AI itself operates as a constitutive element. Conceptualizing AI as a nested complex system facilitates a paradigm shift in how we understand and govern radical technological transformations. This epistemological reorientation brings to light a fundamental imperative: the emergence of powerful technologies demands the activation of our most sophisticated forms of knowledge—not only about the current state of the world but also about how we produce, validate, and manage that knowledge. Such mobilization serves a dual function: addressing pressing global needs while also preventing or mitigating the impacts of potential crises. Yet this task must be undertaken with a clear recognition of its inherent limits. The eco-techno-social world we inhabit is characterized by irreducible complexity, such that no matter how advanced our epistemic tools become, a domain of indeterminate futures will persist beyond the reach of predictive certainty (Rescher, 1998). Before turning in Section Four to the more concrete implications of this conceptual shift for policymaking—at both theoretical and practical levels—the next section offers a brief examination of selected regional AI governance frameworks through the lens of what might be termed resilience analysis. This approach, inspired by a modeling practice in complexity science, evaluates the robustness of a system feature when key parameters are varied or when alternative modeling assumptions are introduced. By applying such analysis to AI governance, we aim to uncover latent vulnerabilities, self-reinforcing mechanisms, and potential leverage points within existing regulatory ecosystems. This serves a dual purpose: first, to assess the extent to which current governance models meaningfully integrate complexity-informed principles; and second, to generate insights into how these frameworks might adapt—or fail to adapt–in the face of novel, emergent challenges posed by AI's dynamic evolution and cross-border impacts. Building on the conceptual foundations discussed above, we now examine how selected governance frameworks reflect—or fall short of—a resilience-informed perspective.

## 4 Governance frameworks through resilience analysis

Global approaches to (AI) regulation currently diverge, reflecting distinct cultural, political, and economic paradigms across regions. The European Union (EU) has established itself as a significant force, particularly with its General Data Protection Regulation (GDPR) influencing international data privacy norms and its risk-based AI Act setting comprehensive standards for AI governance and other technologies. This contrasts markedly with the United States, which employs a more fragmented, sector-specific regulatory model lacking a unified federal standard, leading to inconsistencies and uncertainty further complicated by shifting political administrations. Meanwhile, nations such as China adopt strategies that seek to balance state oversight with market-driven innovation. While is not the main objective of this conceptual paper, we now briefly show how the ideas developed so far can be helpful to further scrutinize enacted and proposed legislation with respect to global systemic resilience. Taking previous legislation as a starting point, data privacy legislation constitutes a checkpoint in the legal trajectory toward effective AI governance, insofar as different kinds of data are the point of departure for most AI pipelines, and have historically constituted a mean for states to gain knowledge and power over populations (Wiggins and Jones, 2023). The European Union's General Data Protection Regulation (GDPR) constitutes a fundamental framework that significantly impacts international norms pertaining to data protection and privacy within the realm of artificial intelligence applications (Matai, 2024). In contrast, the United States adopts a sector-specific regulatory approach, characterized by disparate levels of oversight at both federal and state jurisdictions (Chun et al., 2024). Asian nations, exemplified by China, have instituted regulatory measures that adeptly reconcile state oversight with the imperative for market innovation (Alfiani and Santiago, 2024). These different views on how to conceptualize and enforce data privacy has significantly shaped each region's AI regulatory scheme. Understanding this base is important, not only insofar as they function as a base for future laws and policy decisions, but also as it highlights how different global players think about the very nature of data, the raw material needed for most AI applications. The EU's approach to AI features a comprehensive framework that ensures uniform rules across member states, emphasizing transparency, accountability, and individual rights, such as data access, rectification, and erasure. Furthermore, the EU AI Act, effective from August 2024, adopts a risk-based regulation model, banning high-risk applications like social scoring and real-time biometric identification. It also integrates data privacy with AI governance, enhancing transparency by requiring individuals to be informed when interacting with AI and mandating documentation of algorithms and decision-making processes.^3^

Considering the expandability of the regulatory scheme, The EU will have additional regulations coming into effect in, like the The EU Data Act which will introduce new rules for data access, sharing, and portability for connected and IOT devices. While not free of problems, the EU act is a step forward in terms of increasing the resilience of the whole AI ecosystem. It also exemplifies a good implementation of the principles on AI by the OECD, proposed in 2019 (Yeung, 2020) and updated in 2024 (see also OECD, 2023, 2024), an update that can also be considered as increasing the systemic resilience of both the policy body and the system it attempts to modulate. In this direction, the change to consider “outcomes” rather than mere “outputs” of AI systems already remarks the broad implications automatic decision-making can have and that permeates to the extent and kind of accountability that needs to be encoded in the institutional framework. While the European Union's stringent regulations often establish a high standard for AI governance, other regions emphasize innovation and flexibility, shaped by their unique socio-economic contexts. In stark contrast, the United States presents a fragmented approach to data protection and privacy, lacking a unified federal standard for the processing of personal information in the context of algorithmic development. This gap in comprehensive federal regulation similarly extends to artificial intelligence, with proposed legislation aiming to address the legal responsibilities of AI developers, mandates for transparency, provisions for AI surveillance, and protections for individuals' rights regarding automated decision-making processes.

As these debates evolve, businesses are increasingly urged to proactively adapt their data governance strategies—not only to mitigate risks and ensure compliance with a growing patchwork of state-level regulations, but also to navigate the broader uncertainty surrounding federal oversight, particularly in light of the Biden administration's proposed AI bill being replaced, a development that has sparked both domestic friction and geopolitical tensions. The Trump administration criticized Biden's AI Executive Order, describing it as imposing “unnecessarily burdensome requirements for companies developing and deploying AI,” which hindered “the private sector's ability to innovate in AI by subjecting it to government control”. According to Trump, this approach created “harmful barriers to America's AI leadership.” In contrast, Trump's replacement Executive Order 14,179 adopts a markedly different stance, setting U.S. policy to “sustain and enhance America's global AI dominance to promote human flourishing, economic competitiveness, and national security.” It emphasizes that American AI development “must remain free from ideological bias or engineered social agendas.”^4^

This policy shift reflects a broader commitment to creating an environment where unrestricted innovation can thrive without excessive regulation, positioning the U.S. as a global leader in AI technology. The approach seeks to ensure that ethical considerations are aligned with societal values, though these values are often interpreted through the lens of partisan politics. From a global resilience perspective, this shift in focus represents a significant setback. The prioritization of national interests over global cooperation risks undermining the collaborative efforts needed to tackle the complex challenges posed by AI. It also hampers the global dialogue essential for developing comprehensive frameworks that can guide the responsible development and deployment of powerful technologies—technologies that, for some states, are seen as potential tools for asserting global dominance through either military applications or economic growth. However, this shift also presents an opportunity to forge innovative conceptual frameworks that bridge the gap between national priorities and international collaboration. Fostering an environment where shared knowledge and best practices can thrive to ensure AI benefits all of humanity focusing on the kind of values that drive both policy and technological designs is a means toward more systemic resilience. The challenge, then, lies in balancing these diverse approaches to establish a cohesive global framework of guidelines, perspectives, and values that ensures ethical AI development while fostering both technological and conceptual innovation. As AI technologies continue to evolve, sustained dialogue among stakeholders and adaptive regulatory strategies will be essential to confront the dilemmas that innovation inevitably generates within societies. In the following section, we further elaborate on key conceptual issues that must be addressed and systematically encoded into both technological development and regulatory schemes. These considerations are critical for all stakeholders and point toward viable routes for implementation.^5^

## 5 The challenge of designing resilient institutions

By integrating the principles of complexity science, we acquire a more robust conceptual framework for addressing the multifaceted challenges and opportunities posed by general-purpose technologies. This integration is grounded in explicit philosophical commitments that guide both the modeling and governance of complex systems. It enables the alignment of AI development trajectories with articulated societal needs by foregrounding the importance of modeling material and informational flows as interdependent elements within broader systemic dynamics. Adopting this perspective not only reinforces the theoretical underpinnings of AI governance but also provides practical analytical tools. These tools allow for a more nuanced understanding of the intricate interdependencies characterizing socio-technical systems, the emergent behaviors that arise from their interaction, and the capacity to strategically harness technological innovation in pursuit of normatively desirable societal outcomes within an increasingly interconnected global landscape. A cybernetically inspired epistemic framework—centered on information flows, feedback mechanisms, and adaptive responses as constitutive features of complex systems—provides a compelling paradigm for the design of governance structures capable of co-evolving with the technologies they seek to regulate. This framework is particularly salient for AI governance, where iterative feedback and adaptive capacity must be prioritized over rigid, prescriptive rule-sets that risk obsolescence in the face of rapid technological change. By foregrounding uncertainty and institutional learning, such an approach enables governance architectures to remain responsive and robust. Within this context, resilience should be seen as a core design principle embedded in the structure of regulatory systems. This imperative calls for mechanisms that support functional redundancy and adaptive capacity, thereby safeguarding systemic integrity in the face of component failures or unforeseen disruptions. While such dynamics are often observable in micro-social contexts—for instance, when individuals in small groups assume new roles under duress—scaling them up demands sophisticated infrastructures for parallel information processing and distributed decision-making. These collective epistemic processes, however, hinge on the existence of shared interpretive frameworks that clarify what is at stake, alongside robust communicative architectures capable of both generating and sustaining such understanding. In the final analysis, the feasibility of effective and resilient governance in any complex system rests upon a critical engagement with the epistemological dimensions of political problems—dimensions that are themselves irreducibly complex, normatively contested, and shaped by power-laden dynamics Critically examining the values that underpin both the design of AI systems and the decision-making processes within associated policy frameworks—including the institutions responsible for their enforcement—is essential for addressing the normative political challenges that characterize our increasingly complex societal landscape. As AI becomes more deeply integrated into social practices, the values embedded in its architecture and deployment exert far-reaching influence: shaping collective norms, mediating power relations, and redefining the contours of individual rights. Embracing a complexity perspective illuminates the multifaceted, dynamic interactions between technology, culture, and governance structures. This underscores the necessity for inclusive, deliberative processes that integrate diverse perspectives, while also highlighting the imperative of institutional agility—the capacity to adapt to, or even proactively shape, the trajectories of technological development (Mazzucato, 2013). Such an approach fosters a more nuanced engagement with the ethical implications of emerging technologies and supports the co-creation of governance mechanisms that reflect shared values. By promoting collaborative efforts oriented toward human welfare and democratic legitimacy, it helps ensure that technological innovation remains a means of advancing the common good rather than deepening existing social and economic inequalities. The epistemological dimension of AI governance requires an analytic scope that extends beyond the internal mechanisms of discrete AI systems to include the broader ecosystems in which they are embedded—most notably, digital platforms that increasingly rely on AI technologies. This expanded perspective is essential, as AI systems are not merely technical tools but are becoming influential epistemic agents within complex networks of decision-making and regulation. Accordingly, effective governance must move beyond a narrow focus on transparency, explainability, and human oversight at the level of individual applications—though these remain necessary baseline conditions—to embrace a systemic understanding of how knowledge is produced, mediated, and operationalized within AI-driven environments. Such measures, while necessary, are insufficient on their own. AI systems—often functioning as opaque “black boxes” and operating at unprecedented scale due to automation—have the capacity to reshape processes of knowledge production, influence perception, and subtly steer decision-making across all levels of society. This dynamic risks undermining democratic accountability by diffusing responsibility and obscuring causal chains of influence. Recognizing AI as embedded epistemic agents thus demands a governance framework that goes beyond technical fixes. It must actively embed normative ethical commitments into design and deployment practices, foster inclusive deliberation, and institutionalize mechanisms to integrate marginalized perspectives into the development and regulation of AI. Only such a multidimensional and ethically anchored approach can counteract the systemic risks posed by these technologies and ensure their alignment with democratic values. The epistemological framework advanced in this paper seeks to engage directly with the intrinsic uncertainties upon which technological innovation both relies and expands. Central to this endeavor is the establishment of clear normative guidelines and the cultivation of open, critical dialogue. The objective is to foster an environment in which epistemic accountability—understood as the responsibility for the knowledge claims embedded in, generated by, and made actionable through AI systems—is not merely aspirational but foundational to their development. This form of accountability entails aligning technological trajectories with articulated societal values and democratic deliberation, guiding innovation toward the public good. Achieving this, however, requires a shift in the dominant epistemological assumptions that underpin both design and governance practices. It also demands a critical interrogation of the political configurations that AI enables and reinforces, as these shape not only what can be known but who has the authority to know and decide. The political dimension of AI governance inherently engages with questions of power, democracy, legitimacy, and the social constitution of technology. From a social constructionist perspective, AI systems are not neutral tools but sociotechnical artifacts—shaped by and reflective of the social, cultural, and institutional contexts in which they are developed. These systems invariably embed and may amplify the values, assumptions, and priorities of their designers, thereby reproducing and potentially entrenching existing power asymmetries. Effective governance, therefore, must critically engage with these dynamics, not merely through ethical oversight but by institutionalizing mechanisms for genuinely inclusive participation. This entails ensuring that those most affected by AI systems—often the least empowered—are meaningfully involved in shaping their development, deployment, and regulation. Yet, as previously discussed, current global governance initiatives remain highly fragmented, frequently dominated by the geopolitical strategies of powerful states and the economic interests of large technology firms, limiting the scope for equitable and democratic oversight.

This concentration of technological capacity and control largely within a few corporate entities, whose commercial imperatives may prioritize profit over broader societal benefits, raises profound concerns regarding democratic legitimacy and equitable representation—particularly as digital platforms mediated by AI become increasingly central arenas for political discourse and public life. Addressing this requires governance structures that actively counterbalance concentrated power and ensure AI development aligns with democratic principles and public welfare.^6^ The political-epistemological cybernetic framework proposed in this paper addresses these concerns by emphasizing the need for mechanisms that foster inclusive participation across regions and socio-economic divides. It advocates for redistributing decision-making power within governance structures, ensuring accountability for both private and public AI developers, and maintaining democratic oversight while promoting innovation. Due to space constraints, we cannot provide a comprehensive analysis of the governance structures of technologies that exemplify this perspective, such as open-source software development. However, the reader may find it useful to consider open-source software as a valuable model for community-led governance of complex systems. It represents an accessible means for end-users to engage with digital tools in a more sovereign manner, offering greater transparency over the software stack in use. The philosophical problem, therefore, revolves around how epistemic and political authority is constituted within AI systems. It questions which knowledge frameworks are privileged or marginalized in their design and how the resulting technological artifacts influence our collective understanding of social reality. This recursive relationship between knowledge production and technological embodiment raises critical questions about the conditions necessary for creating equitable and epistemically sound AI governance structures. The governance of artificial intelligence requires the establishment of robust epistemological frameworks that address several interconnected imperatives. First, principles of transparency and explainability must be embedded in the architectural design of AI systems, incorporating mechanisms that make the underlying inferential processes accessible to diverse epistemic communities. This accessibility goes beyond technical disclosure, necessitating the translation of algorithmic operations into conceptual frameworks that are intelligible to a range of stakeholders with varying expertise and interpretive resources. This principle also extends to how terms and conditions of digital services are disclosed.

Second, several forms of epistemic pluralism must be valorized through the deliberate incorporation of heterogeneous knowledge modalities and disciplinary backgrounds. This entails moving beyond the privileging of mere technical rationality to embrace ethical, social, and cultural forms of knowing that may illuminate dimensions of AI development and deployment otherwise obscured by purely instrumental perspectives. The integration of these diverse epistemic standpoints serves as a corrective to the potential narrowness of purely technical epistemologies.

Integrating governance and compliance considerations into the early stages of AI system development transforms epistemological concerns from mere afterthoughts into core design principles. This proactive epistemological stance creates conditions that foster trust and accountability throughout the technological lifecycle, effectively addressing the fundamental challenge of epistemic opacity that threatens the social legitimacy of artificial intelligence systems.

The pressing question, however, is how to effectively create mechanisms and new institutions that can operationalize this framework. AI governance presents unique challenges that require a rethinking of institutional design. Traditional governance structures, which tend to be linear and rigid, are ill-equipped to address the dynamic and multifaceted nature of AI systems. Instead, institutions must be designed with resilience as a foundational principle, emphasizing adaptability, inclusivity, and ethical oversight. This section explores the key factors in developing resilient institutions for AI governance, drawing on insights from complexity science and public policy.

Resilience-oriented design begins with the recognition that AI systems operate within complex socio-technical networks, characterized by interdependencies that amplify both the risks and benefits of technological advancements. To effectively govern this complexity, institutions must adopt decentralized and distributed governance frameworks (Morçöl, 2023). These frameworks distribute oversight among a diverse array of stakeholders—including governments, private enterprises, civil society organizations, and academia—enhancing inclusivity and fostering innovative solutions that reflect the varied needs and values of global communities (Bitas and Harjani, 2020).

The principle of modularity is central to resilience-oriented institutional design. Modular institutions are defined by their capacity to add or remove components as needed, allowing them to adapt to rapidly evolving technological landscapes. This adaptability is crucial for AI governance, where new risks and opportunities emerge continuously. By incorporating modularity, institutions can better withstand shocks while maintaining functionality, thus embodying the dual meanings of resilience: both resistance and adaptability (Pečarič, 2020). Ashby's law of requisite variety, a foundational principle in cybernetics, underscores the necessity for institutions to match the complexity of the systems they seek to govern, ensuring that regulatory measures remain effective in the face of technological advancements (Young, 2017). Effective governance, therefore, requires attention to the dynamic feedback mechanisms and adaptive strategies that underpin both technological innovation and policy development.

The interaction between AI technologies and societal structures—both formal and informal—creates a recursive loop where each continuously influences and reshapes the other. As stakeholders engage with AI systems, their inputs and reactions contribute to an evolving landscape of capabilities and expectations. Therefore, policies must not only address the current technological landscape but also anticipate future trajectories by integrating adaptive governance frameworks that are flexible enough to accommodate rapid changes while maintaining resilience against uncertainties.

Effective governance also requires a deep understanding of AI systems' complexities. Regulatory frameworks must capture the full scope of these technologies, embracing their intricacies rather than oversimplifying or abstracting away their defining properties. Methodologies such as scenario analysis, simulation exercises, and empirical experimentation enable regulators to foresee and mitigate potential risks. For instance, simulation exercises can model AI behavior under various conditions, identifying vulnerabilities and informing regulatory strategies (Hadfield and Clark, 2023). These approaches not only enhance the safety and reliability of AI systems but also foster innovation by providing clear, adaptive guidelines.

Collaboration among stakeholders is another critical element of resilient governance. AI systems interact with a broad spectrum of societal dimensions, requiring input from experts across various disciplines. Interdisciplinary advisory boards, which integrate insights from technology, ethics, law, and the social sciences, create a collaborative space for developing comprehensive policies. However, this collaboration must carefully navigate the tension between transparency and competitive advantage, as industry stakeholders may be reluctant to share information in order to protect proprietary interests (McCarty, 2017). Building trust among stakeholders is crucial to overcoming these challenges and ensuring the effectiveness of regulatory measures.

Resilience-oriented institutions must also prioritize human values and societal wellbeing. Equity, transparency, and accountability should underpin all aspects of AI governance. This involves designing AI systems that align with societal norms and ethical standards, fostering trust among users and stakeholders. As Frenken (2006) observed for other cases of technological innovation, the unequal distribution of technological capabilities exacerbates social and economic inequalities, creating new forms of exclusion. Addressing these disparities requires a value-driven design perspective that integrates local contexts and cultural considerations into AI development and deployment. While not free of conceptual and operative problems, at the moment Human Rights should be interpreted as the most significant guide for shared societal values to guide all fronts of AI developments, including oversight mechanisms (Aizenberg and Van den Hoven, 2020; Montemayor, 2023; Parra-Dorantes, 2024; Renieris, 2023).

Education and continuous assessment are integral to the resilience of governance frameworks. Policymakers and regulators must stay abreast of rapid technological advancements, ensuring that governance measures remain relevant and effective. This requires ongoing education for stakeholders, from technical experts to the general public, fostering a more informed and engaged society. Feedback mechanisms, such as monitoring and reporting systems, enable institutions to evaluate the efficacy of governance strategies and identify areas for improvement (Smuha, 2021).

Finally, fostering a culture of experimentation is crucial for resilience-oriented governance. Institutions should create environments where innovation is encouraged, and failures are viewed as valuable learning opportunities. This approach embodies a form of scientific rationality that functions effectively through feedback and control mechanisms, advancing knowledge despite the paradox that each discovery leads to exponentially more questions—highlighting the more we know, the more we realize how much remains unknown. This mindset promotes adaptive, proactive problem-solving, enabling organizations to navigate the complexities of AI systems effectively. By embracing resilience under uncertainty as a guiding principle, institutions can develop governance frameworks that not only address immediate challenges but also ensure long-term sustainability and equity amid rapid technological change. This approach requires policymakers and scientists to be literate in the nature of scientific inquiry and the uncertainties it entails (Douglas, 2009; Kotsis, 2024; Lane et al., 2011; Ludwig, 2001).

As this brief presentation has shown, designing resilient institutions for AI governance requires a multifaceted approach that draws on complexity science, fosters interdisciplinary collaboration, and remains grounded in a steadfast commitment to human values. By embedding adaptability, inclusivity, and ethical oversight as core principles, such institutions can more effectively navigate the dynamic challenges posed by an AI-driven society while continuing to foster innovation and promote collective wellbeing. This approach not only strengthens the efficacy and legitimacy of governance frameworks but also helps ensure that AI development remains aligned with the principles of equity and sustainability—contributing to a more just and resilient global future.

Within current scholarship on public policy and governance, some of the most promising approaches aligned with our proposal are the concepts of polycentric and adaptive governance (Folke et al., 2005; Garmestani and Benson, 2013). The latter can be understood as a specific form of polycentric governance, which offers a complex, multi-layered framework for collective decision-making and institutional organization. Originally conceptualized by Vincent and Elinor Ostrom of the Bloomington School of Political Economy, this model has gained increasing traction as a means of understanding how societies confront complex and interdependent social challenges across diverse domains. At its core, polycentric governance is characterized by the coexistence of multiple centers of decision-making authority, each formally autonomous but operating within a broader, interdependent system. According to Ostrom, Tiebout, and Warren's seminal 1961 definition, “polycentric connotes many centers of decision-making which are formally independent of each other. Whether they actually function independently, or instead constitute an interdependent system of relations, is an empirical question in particular cases” (Ostrom et al., 1961, p. 831). This concept stands in direct contrast to monocentric governance, where a single center of authority makes all decisions in a top-down fashion. Instead, polycentric systems feature multiple decision-making units that maintain some degree of autonomy while still interacting with each other in meaningful ways.

The essence of polycentricity emerges when multiple centers of decision-making take one another into account through various forms of interaction—ranging from cooperation and competition to mechanisms of conflict resolution. When these interactions yield stable and predictable patterns of behavior that enable the system to function coherently in the absence of a central authority, such governance can be said to embody resilience. In this context, resilience becomes a functional concept: a heuristic for achieving outcomes that, in retrospect, can be interpreted as aligned with the normative values that initially motivated the intervention. Coordination, then, is not imposed from above through centralized commands but is instead facilitated by the sharing of values—values that have been institutionally encoded into a governance matrix. Within this matrix, both human and artificial agents act as epistemic participants, guided by heuristics that are accessible and actionable, and which reflect the training and socialization processes through which these agents have acquired their practical knowledge.^7^

## 6 Conclusions

The integration of artificial intelligence into global governance systems signals a transformative juncture, marked by both unprecedented opportunities and formidable challenges. This paper has examined the entangled dynamics of AI, systemic resilience, and complexity science, highlighting the multifaceted character of AI as a socio-technical phenomenon. While AI offers the capacity to enhance operational efficiency and reconfigure decision-making architectures, its widespread deployment also necessitates critical examination. Without careful governance, these systems risk reinforcing ethical blind spots, deepening structural inequalities, and generating unforeseen consequences that may undermine their legitimacy and long-term stainability.

At the heart of this analysis lies the concept of systemic resilience—a foundational lens for understanding how AI can be mobilized to confront global challenges while preserving adaptability, inclusivity, and equity. Drawing on principles from complexity science and the epistemological orientation it entails, policymakers and institutions are better equipped to navigate the uncertainties and interdependencies that characterize contemporary AI systems. This perspective calls for a departure from reductionist approaches in favor of holistic governance strategies that attend to emergent behaviors, recursive feedback loops, and the shifting boundaries that shape socio-technical assemblages.

One of the paper's key conclusions is that leveraging AI effectively requires not only technological innovation but also a commitment to inclusivity and ethical oversight, by means of values encoded in decision assessment procedures. Governance frameworks must therefore prioritize certain values—such as equity, transparency, and accountability—to ensure that AI technologies do not exacerbate existing inequalities or create new forms of marginalization. Inclusive policies that democratize access to AI infrastructure and meaningfully incorporate diverse epistemic perspectives are essential to cultivating a more just and equitable global governance architecture.

This in turn requires a thorough philosophical reinterpretation and ongoing scholarly work to better understand how technological advances reshape what such concepts mean and how they align to other values. Moreover, fostering resilience in AI governance calls for institutional adaptability and collaboration. By embracing modularity and decentralized decision-making, institutions can remain agile in the face of rapid technological advancements. Collaborative approaches that integrate interdisciplinary expertise and stakeholder input enhance the robustness of governance strategies while addressing the ethical and practical complexities of AI integration.

Finally, the role of education and continuous assessment is indispensable. Policymakers, industry leaders, and civil society actors must remain attuned to the evolving capabilities and implications of AI in order to ensure that governance frameworks retain their efficacy and relevance. Cultivating a culture of learning and experimentation allows institutions to develop adaptive policies that are responsive to emerging challenges, while remaining grounded in democratic values and long-term visions of sustainability and social justice.

The transformative potential of AI for global governance is immense, yet its responsible realization hinges on the deliberate design and implementation of resilient, inclusive, and ethically grounded institutional frameworks. By integrating insights from complexity science with innovative governance practices, it becomes possible to harness the capabilities of AI while safeguarding equity, accountability, and systemic stability in an increasingly interconnected and uncertain world.

## Footnotes

^1^Some of the most concerning examples of AI exacerbating inequalities appear in healthcare settings. Research conducted on intensive care unit datasets revealed that AI models for resource allocation induced significant inequalities, with disparity levels ranging from 2.45% to 43.2% for non-White compared to White patients (Wu et al., 2022). Even more troubling, these models sometimes exacerbated existing inequalities by more than nine times their original levels, old-age being a variable that seems to consistently introduce harmful bias (Stypińska and Franke, 2023). Such algorithmic discrimination can have life-or-death consequences when deployed in critical care settings. Candidate screening for job applications is another area in which human oversight has been enforced in many jurisdictions. Without such oversight, AI-powered hiring tools trained on historical employment data may replicate patterns of racial discrimination, creating technological barriers to equal employment opportunity (Clavell and González-Sendino, 2024). The automation of hiring processes through potentially biased algorithms risks scaling discrimination to unprecedented levels. At a judiciary level, perhaps the most known case of inequality that continues to resonate is the use of COMPASS for parole decisions in the US, as reported by ProPublica in 2016 (see Rudin et al., 2020).

^2^Ludwig was particularly attuned in suggesting a fundamental rethinking of how science (i.e., scientists) engage with wicked problems. Two brief quotations illustrate his recommendations that scientists “must be prepared to share their advisory and decision-making roles with a variety of interested parties and participate with them on an equal footing” (Ludwig, 2001, p. 758) and that they “be prepared to admit their limitations and to acknowledge the role that values play in their recommendations” (Ludwig, 2001, p. 763.). We thank an anonymous reviewer for pointing out this important paper. On the topic of values in science, our views have been shaped considerably by Lacey (2005) and Longino (2020).

^3^The EU AI Act relies on harmonized standards to define technical requirements, though this approach has been critiqued for potentially undermining democratic accountability (Gamito, 2024; Gamito and Marsden, 2024). This is an important topic we cannot fully address here, but we note that it forces us to rethink what individuals and institutions have to be able to know and understand about technological schemes. See also section four below.

^4^All quotations in this paragraph have been attributed to Donald Trump by The White House: https://www.whitehouse.gov/fact-sheets/2025/01/fact-sheet-president-donald-j-trump-takes-action-to-enhance-americas-ai-leadership/.

^5^A further comparison of AI current enforced and proposed regulatory frameworks is beyond the scope of this paper, but two excellent sources can be referred for further discussion. Corrêa et al. (2023) conducts a meta-analysis of 200 governance policies and ethical guidelines for AI usage, identifying at least 17 resonating principles that are prevalent across the dataset, which includes contributions from public bodies, academic institutions, private companies, and civil society organizations worldwide. From the resonating principles we can highlight accountability, beneficence, human rights, pluralism, democratic values, intellectual property, labor rights, fairness, privacy and truthfulness. We discuss some of them in the following section. The findings from the study have been compiled into an open-source database and tool, which can be utilized by researchers, policymakers, and practitioners to better understand and implement ethical AI practices. Similar previous work had been documented by Fjeld et al. (2020). More recently (Arnold et al., 2024) introduces AGORA, an extremely useful dataset that compiles AI-related laws and policies across various jurisdictions, which can be utilized to analyze and compare regulatory frameworks and technological standards for AI governance. AGORA's taxonomy includes aspects such as risks, governance strategies, and application domains, facilitating a comprehensive examination of data privacy laws, ethical guidelines, interoperability, and technical specifications. This resource supports deep analysis of the evolving AI governance landscape, enhancing understanding and compliance efforts.

^6^OpenAI current organizational structure is an interesting exception and a case worthy of more scholarly work; yet it is reportedly in the middle of transitioning to a full for-profit scheme.

^7^The wording here has been crafted to resonate with Herbert Simon's approach to rationality, administrative behavior and artificial intelligence in complex environments (Simon, 1996). Further work in this direction is ongoing to hybridize the complexity perspective here elucidated with Simon's work on bounded rationality and currents trends in polycentric governance (Aguerre et al., 2024; Gadinger and Scholte, 2023; Thiel et al., 2019).
